# Supplementary material for: Natural alleles of LEAFY and WAPO1 interact to regulate spikelet number per spike in wheat
Source: Theor Appl Genet. 2024 Oct 24;137(11):257. doi: 10.1007/s00122-024-04759-x (PMC11502542; doi:10.1007/s00122-024-04759-x)

Supplementary Figure

Figure S1. Alignment of LFY proteins from multiple grass species. The query is the *T. aestivum LFY-B* haplotype 5 (P+/34L/80S). Only the region around the L34P and R80S polymorphisms is presented. *Triticum urartu* is included as the *T. aestivum* donor of the A genome and *Aegilops tauschii* as the donor of the D genome. These two species as well as several other grasses carry the 34P and 80R alleles, which are considered as the ancestral alleles. Underlined is a stretch of eight proline residues that are reduced to seven in haplotype H4 (8 P to 7 P).

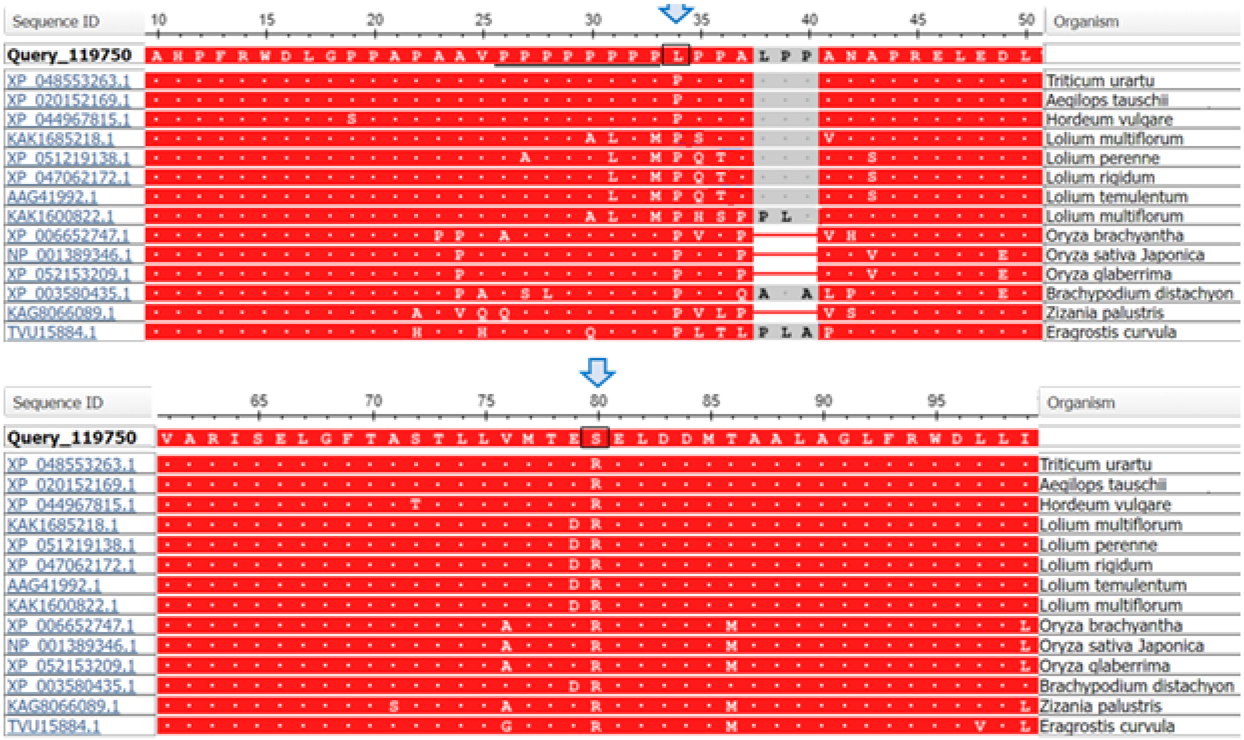

Supplement: Supplementary file 2 — Supplementary file2 (PDF 248 KB) [file 122_2024_4759_MOESM2_ESM.pdf]
